# A Machine Learning-Assisted Liquid Crystal Droplet Array Platform for the Sensitive and Selective Detection of Per- and Polyfluoroalkyl Substances (PFAS) in Water

**DOI:** 10.1021/acssensors.5c00907

**Published:** 2025-09-25

**Authors:** Fengrui Wang, Shiyi Qin, Zhao Yang, Leena M. Edwards-Medina, Benjamin L. Chiu, Claribel Acevedo-Vélez, Christina K. Remucal, Reid C. Van Lehn, Victor M. Zavala, David M. Lynn

**Affiliations:** † Department of Chemistry, 5228 University of Wisconsin−Madison, 1101 University Avenue, Madison, Wisconsin 53706, United States; ‡ Department of Chemical and Biological Engineering, University of Wisconsin–Madison, 1415 Engineering Dr., Madison, Wisconsin 53706, United States; § Department of Civil and Environmental Engineering, University of Wisconsin–Madison, 660 North Park Street, Madison, Wisconsin 53706, United States; ∥ Department of Chemical Engineering, University of Puerto Rico-Mayagüez, Call Box 9000, Mayagüez, Puerto Rico 00681-9000, United States

**Keywords:** machine learning, liquid crystals, neural networks, autoencoder, PFAS

## Abstract

We report a machine learning (ML)-assisted liquid crystal (LC) droplet array platform for the detection of per- and polyfluoroalkyl substances (PFAS) in water. Our approach uses an autoencoder network to process thousands of images obtained from arrays of microscale droplets of thermotropic LCs. The latent space obtained using the autoencoder contains significant information that enables sensitive and selective detection of two amphiphilic PFAS [perfluorooctanoic acid (PFOA) and perfluorooctanesulfonic acid (PFOS)] at concentrations as low as parts-per-trillion (ppt) in ultrapure water, municipal tap water, and simulated river water containing dissolved organic matter. Despite the absence of visually discernible changes in the optical outputs of LC arrays at low PFAS concentrations, this approach accurately predicts their presence, even in water containing interfering molecules. We also demonstrate the use of transfer learning to differentiate between PFOA, PFOS, and PFOA/PFOS mixtures, showcasing the potential for practical environmental monitoring. This platform permits identification of PFOA and PFOS below the maximum contaminant levels (4 ppt) established by the U.S. Environmental Protection Agency. Our approach is compatible with automated printing, treatment, and high-throughput optical and ML analysis and could provide a basis for the development of low-cost sensors to monitor PFAS and other amphiphilic contaminants in real-world water samples.

## Graphical abstract

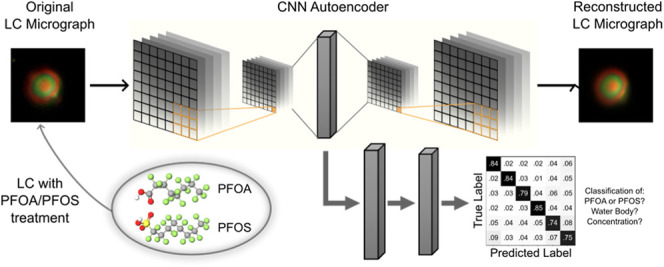

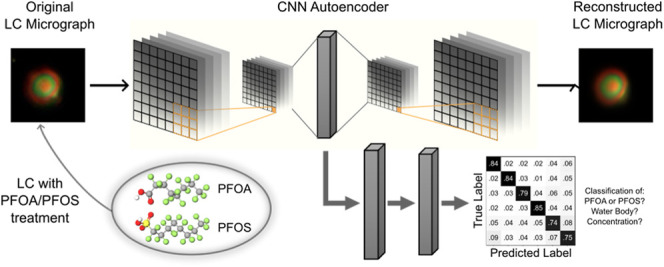

Per- and polyfluoroalkyl substances (PFAS) have unique chemical properties^1–3^ and have been used extensively in a wide range of applications, including consumer products, industrial applications, firefighting foams, food packaging, and various products and materials used in the healthcare industry.^4–9^ Among the thousands of PFAS compounds in use, perfluorooctanoic acid (PFOA) and perfluorooctanesulfonic acid (PFOS) are the most widely studied in various media and environments.^10–12^ Recent toxicological and epidemiological studies have raised significant concerns about these long-chain PFAS compounds,^13,14^ leading the U.S. Environmental Protection Agency (EPA) to establish regulatory maximum contaminant levels (MCLs) of 4 parts-per-trillion (ppt) and non-enforceable maximum contaminant level goals of 0 ppt each for PFOA and PFOS in drinking water in 2024.^15^

In view of these and other concerns, it is becoming increasingly critical to be able to efficiently monitor the concentrations of PFOA and PFOS in many different kinds of municipal, industrial, and environmental water samples. Current methods for PFAS analysis in water samples typically involve some form of sample preparation, such as solid phase extraction (SPE), followed by quantification using liquid chromatography–tandem mass spectrometry (LC–MS/MS).^16^ Although this method enables precise, selective, and sensitive measurements and is currently regarded as the gold standard for the quantification of PFAS in water, the overall process requires specialized facilities, well-trained personnel, and time-consuming and expensive sample preparation and analysis, especially when dealing with low-concentration samples.^17–20^ Furthermore, data accuracy is also restricted by the limit of quantification (LOQ) of individual instruments. Alternative methods of analysis that are sensitive, rapid, cost-effective, and mobile are highly sought after but have been difficult to achieve, at least in part because of the inherent compositional complexity of many real-world water systems.^20–25^

We recently reported a new machine learning (ML) workflow that uses a hierarchical convolutional neural network (CNN) to analyze experimental outputs obtained from microcontact printed arrays of liquid crystal (LC) droplets treated with aqueous analytes.^26^ We demonstrated that the workflow could detect nanomolar-level concentrations of a simple synthetic surfactant (sodium dodecyl sulfate; SDS) and differentiate the presence of multiple surfactants and surfactant mixtures in aqueous solutions. The long-range orientational ordering and birefringence of the surface-bound LC droplets allow these arrays to amplify molecular-level interactions and transduce them into tens, hundreds, or potentially thousands of microscale optical outputs depending on the sizes of the arrays.^27–31^ That past study established that ML can substantially enhance the operational sensitivity and selectivity of LC-based materials to aqueous amphiphiles by identifying and interpreting optical features of LC droplets that are not otherwise discernible to the trained human eye.

Effective monitoring of many PFAS compounds in water currently requires the detection of concentrations at ppt levels. In the future, it may become necessary to detect PFAS at even lower levels. This current study was motivated by this broad challenge and sought to determine whether our ML-assisted LC array platform could have potential as a basis for the sensitive and selective detection and monitoring of these amphiphilic pollutants in water. In this paper, we report the use of an autoencoder neural network to analyze optical responses obtained from LC arrays treated with samples of water to selectively and sensitively detect PFOA and PFOS in various types of water matrices, including ultrapure laboratory (Milli-Q) water, municipal tap water, and samples of simulated river water (RW) containing dissolved organic matter (DOM, a heterogeneous and complex mixture of organic molecules derived primarily from plant and microbial residues that is found in all water bodies, with concentrations ranging from 1 to 35 mg C/L).^32–35^ Autoencoders have the ability to capture complex and subtle features within high-dimensional data. To generate a robust data set for training the autoencoder, we developed an experimental setup that utilizes a robotic printing process to produce tens of thousands of surface-immobilized LC droplets that can be imaged using polarized light as arrays to rapidly generate large quantities of data. This approach permitted the collection of large numbers of LC micrographs, resulting in a data set of over 11,000 droplet images that was expanded to over 225,000 droplet images after 20-fold augmentation. By encoding these LC micrographs into a lower-dimensional latent space, the autoencoder effectively reduced background noise and isolated diagnostic LC droplet optical patterns that are not otherwise visually discernible. We illustrate that even though low concentrations of these analytes (e.g., 1 part per million (ppm), 1 part per billion (ppb), and 1 part per trillion (ppt) PFOA) do not lead to changes in the optical appearances of the LC droplets that are discernible or diagnostic to the trained human eye, the neural network can extract useful information to accurately predict their presence. In the context of potential practical applications of this approach, we demonstrate further that this system can accurately detect and report the presence of ultralow concentrations of PFOA even in complex water matrices such as municipal tap water and simulated river water that contain other potentially interfering molecules, including ions and DOM.^36–38^ Finally, we also demonstrate the ability of the trained autoencoder to facilitate transfer learning, which enables the detection of PFOS at levels as low as 3.5 ppt and can differentiate between samples containing PFOA or PFOA/PFOS mixtures. Overall, our results offer a proof-of-concept demonstration of the ability of this system to sensitively and selectively detect these critical environmental pollutants in complex media. Our results also showcase the generalizability of our system for the detection of other PFAS or environmental pollutants under more complex conditions with the assistance of transfer learning strategies.

## Materials and Methods

### Materials

The nematic thermotropic LC 4′-pentyl-cyanobiphenyl (5CB) was purchased from HCCH Jiangsu Hecheng Display Technology Co., Ltd. (Jiangsu, China). Glass coverslips were obtained from Fisher Scientific (Pittsburgh, PA). An automated *xyz* dipping robot (Riegler & Kirstein GmbH, Potsdam, Germany) was used to prepare the LC droplet arrays. Perfluorooctanoic acid (PFOA) and perfluorooctanesulfonic acid (PFOS) were obtained from Sigma-Aldrich (Milwaukee, WI). [^13^C_8_] PFOA and [^13^C_8_] PFOS surrogates used in this study were obtained from Wellington Laboratories Inc. (Guelph, Canada). HPLC-grade methanol (MeOH, ≥99.9%) was purchased from Fisher Chemical (Waltham, MA). Reagent-grade ammonium hydroxide (30%) was purchased from JT-Baker Inc. (Phillipsburg, NJ). LC–MS-grade ammonium acetate was purchased from Honeywell Research Chemicals (Muskegon, MI). Weak anion exchange (WAX) cartridges were purchased from Waters Corp. (Milford, MA). Ultrapure water (18.2 M Ω·cm) was obtained using a Milli-Q water system. Upper Mississippi River natural organic matter (DOM; RO/ED isolation) was purchased from the International Humic Substances Society (IHSS); details of the DOM can be found on the IHSS Web site.^39^ Municipal tap water was collected from a laboratory sink tap in Engineering Hall at the University of Wisconsin–Madison (Madison, WI, USA); samples from the same tap were used in all studies. Simulated river water was prepared by dissolving the DOM in municipal tap water to reach a final concentration of 10 mg of dissolved organic carbon per liter of simulated river water (10 mg C/L), and the pH was adjusted to 7.0.^35^ All materials were used as purchased without further purification unless noted otherwise.

### General Considerations

Bright-field and polarized-light microscopy images were acquired using an Olympus IX71 inverted microscope (Waltham, MA) equipped with cross-polarizers (Olympus analyzer slider IX2-AN and condenser attachment IX-LWPO). Fields of view were recorded using an OPTO-EDU (Beijing, China) eyepiece camera model A59.2211 connected to a computer and controlled through ImageView imaging software, version A30.2201.

### Microcontact Printing of LC Droplet Arrays

PDMS stamps consisting of an array of pillars (50 μm square pillars with 50 μm separating distance between each pillar) were prepared as previously described.^40^ All LC arrays used in this study were printed using a single master stamp and an *xyz* robot to standardize production and reduce the potential for variation that could result from manual stamping protocols used in that past study. The stamp was rinsed with EtOH and then attached to the arm of the *xyz* dipping robot for printing of the LC droplet arrays. Inking of the stamps was achieved by first spreading a 5 μL droplet of 5CB on a clean glass substrate using another glass plate to produce a thin layer. The stamp was moved by the robot arm and then lowered to gently touch the liquid crystal-spread surface for 5 s. The stamp was then quickly raised and moved to a freshly ethanol-washed glass cover slide for printing. The stamp was then lowered into contact with the cleaned substrate and then held in that position for 10 s. The stamp was then removed by raising the robotic arm. The stamp was fully rinsed with EtOH between prints. The quality of the droplet arrays was inspected using an optical microscope to ensure uniformity in droplet sizes and assess print fidelity. Different aqueous surfactant solutions (20 μL) were then carefully introduced to the printed LC arrays using a micropipette as described previously,^26^ and LC droplet arrays were imaged using an optical microscope and cross-polarized light. All analyte treatment and image collection experiments were performed by a single common researcher in a small, fully enclosed, windowless, and air-conditioned/climate-controlled room that was fully darkened during image collection. Images of arrays treated with each analyte were acquired using a single microscope using the same settings and in random order with each array treated with multiple analytes over multiple days of experiments and then pooled to reduce the potential for biases from any potential variations in array structure, imaging conditions, or other experimental factors on any given day. Each acquired image contained multiple droplets and was then cropped into smaller images of individual droplets that were randomly assigned to training and validation sets, as described in greater detail below.

### Preparation of PFOA and PFOS Solutions

Stock solutions of 0.1 mg/mL PFOA were made by dissolving the purchased chemicals directly in different water samples. The 1 ppm, 1 ppb, and 1 ppt PFOA samples were then prepared by serial dilution. The stock concentrations of PFOA and the accuracy of the serial dilutions were confirmed using LC–MS/MS (see Table S2 for the experimentally determined concentrations). The PFOS solutions were prepared by serial dilution of a 1.3 ppb PFOS stock solution that was previously quantified using LC–MS/MS.

### Quantification of PFOA and PFOS Solutions Using LC–MS/MS

#### Sample Preparation

A previously established LC–MS/MS method was used to quantify the amount of PFOA and PFOS in the following samples:^9^ stock 0.1 mg/mL, 1 ppm, and 1 ppb PFOA; stock 1.3 ppb PFOS; Milli-Q water; and municipal tap water. To maintain sample integrity, all samples were stored at 4 °C until they were ready for analysis. Samples were allowed to equilibrate to ambient room temperature on the day of analysis. All samples with different spiked levels of PFAS were prepared and analyzed using a systematic solvent dilution approach. For high spiked levels (100 and 1 μg/mL), a 1 μL aliquot was transferred to a vial containing 999 μL of a solvent mixture composed of 80% HPLC-grade MeOH and 20% ultrapure water, achieving a dilution factor of 1000. A secondary dilution for the 100 μg/L sample involved transferring 10 μL of the primary dilution to a new vial containing 990 μL of the same solvent mixture, effectively diluting the sample by an additional factor of 100. The sample spiked at 1 ng/mL was diluted using HPLC-grade MeOH to achieve a final composition of 80% MeOH/20% water. For the final analysis, 96 μL of the prepared diluted solution was combined with 4 μL of a 200 μg/L surrogate to ensure the quantitative accuracy of the analysis. Matrix samples, including tap water and ultrapure water, were prepared via SPE using a Supelco Visiprep manifold equipped with WAX cartridges, following a published method from a former study.^9^ Volumes are listed in Table S3.

#### LC–MS/MS Analysis

PFOA and PFOS were quantified on an Agilent 1260 LC system equipped with an Agilent 6460 triple quadrupole mass spectrometer. Details for parameters used in chromatographic analysis can be found in a former study.^9^

#### Analysis and Quality Assurance/Quality Control (QA/QC)

Method blanks, solvent (i.e., analytical) blanks, and calibration verifications were included as quality control samples. Mass-labeled surrogate recoveries were used to provide insight into the matrix effects and account for analyte losses during the extraction for target PFAS analysis. Additionally, for each compound, transitions (quantifier and qualifier) were monitored where transitions are possible, as indicated in Table S1. Each analytical run included six calibration standards (0.1, 0.5, 1, 5, 10, and 20 ng/mL), laboratory extraction blanks, instrument blanks, and midpoint QCs (0.1 and/or 5 ng/mL). All quality control samples except for instrument blanks were spiked with 4 μL of 200 μg/L mass-labeled surrogates. The final composition of all quality control samples was the same as that of unknown samples (80% methanol/20% water). The limit of quantification (LOQ) for PFOA (0.1 ng/mL, ppb) and PFOS (0.1 ng/mL, ppb) prior to SPE was determined as the lowest calibration curve point where the calculated concentration was ±30% of the true concentration; LOQs as low as 0.03 ppt were achieved after SPE (Table S3). QCs were performed by injecting a standard after every 10 unknown injections (alternating between 0.1 and 5 ng/mL), and sample data were accepted only if QCs were ±30% of the true value. Calibration curves were fit with regression equations (*R* ^2^ > 0.99) and used to quantify analytes in unknown samples. Every sample was quantified using an isotope dilution method. The determined concentrations of the tested samples are summarized in Table S2, and the serial dilution process was determined to be accurate. Averaged recoveries (%) of mass-labeled surrogates (PFOA and PFOS) in all analyzed samples and laboratory extraction blanks were monitored, with a range of 115–117%.

### Droplet Cropping and Image Augmentation

We used a Canny edge detection method with a Gaussian filter to identify potential droplet image crops from whole images containing many droplets. These droplet image crops were selected by distribution-determined size cutoffs, eliminating any irregularities and automating whole image processing. Once cropped, each droplet image was augmented 20-fold to enhance the data set via techniques such as random shearing, rotation, and flipping. In total, 225,700 images (11,285 raw micrographs with augmentation) were used to train our models.

### Model Implementation

The autoencoder network consisted of an encoder and a decoder. The encoder used a CNN architecture featuring three 2D convolutional layers with average pooling layers, batch normalization, and ReLU activation. The decoder was designed with the same architecture but in a transposed manner. The latent space comprised 128 neurons, a size determined through a grid search of this hyperparameter for optimal reconstruction error (evaluated for 32, 64, and 128 neurons). The classifier network included two hidden layers with 256 and 128 neurons, respectively, and a dropout layer (20% dropout rate). The model was implemented using PyTorch (version 1.2.0).

For all classification tasks, we performed a 5-fold cross-validation with stratification based on class labels to ensure that the training and validation sets maintained the same class ratio. Given that the autoencoder was jointly trained with a classifier, the loss function used in this study was a combination of the reconstruction loss ( $L_{recon}$ , mean-squared error) and the classification loss ( $L_{classifier}$ , cross-entropy). To ensure that both loss components contributed equally, we applied dynamic normalization to each loss component. The dynamic normalization was implemented using exponential moving averages (EMA) updated at each epoch. EMA was chosen for normalization because of its ability to smooth out fluctuations and provide a stable estimate of the loss values over time, which is particularly beneficial for handling varying scales of multiple loss components.^41–43^ Specifically, the average reconstruction loss and average classification loss were calculated as follows:

$${\mathrm{L̅}}_{recon}^{\left(t\right)}=\alpha \cdot {\mathrm{L̅}}_{recon}^{\left(t-1\right)}+\left(1-\alpha \right)\cdot L_{recon}^{\left(t\right)}$$

$${\mathrm{L̅}}_{classifier}^{\left(t\right)}=\alpha \cdot {\mathrm{L̅}}_{classifier}^{\left(t-1\right)}+\left(1-\alpha \right)\cdot L_{classifier}^{\left(t\right)}$$

where the smoothing parameter α was set to 0.9, giving more weight to the previous moving average and less weight to the current loss value. These averages were used to normalize the respective losses dynamically and then to compute the combined loss $L_{total}^{\left(t\right)}$ :

$$L_{total}^{\left(t\right)}=\beta_{recon}\cdot \frac{L_{recon}^{\left(t\right)}}{{\mathrm{L̅}}_{recon}^{\left(t\right)}}+\beta_{classifier}\cdot \frac{L_{classifier}^{\left(t\right)}}{{\mathrm{L̅}}_{classifier}^{\left(t\right)}}$$

where the weight parameters β_recon_ and β_classifier_ were equally set to 0.5. The model parameters were optimized using the Adam optimizer with a learning rate of 0.001. For each cross-validation fold, the model was trained for 50 epochs with a batch size of 200. To address class imbalance during neural network training, we applied a weighted loss function to classification loss where weights were computed inversely proportional to class frequencies. Model accuracy was assessed by analysis of correctly predicted classes for pooled (compiled) validation samples during 5-fold cross-validation as well as calculation of macro-averaged F1 scores for each classification task. To estimate model error, we also report the standard deviation of the F1 score when comparing values computed for each fold separately.

## Results and Discussion

The work reported here builds upon the general experimental and ML-assisted conceptual framework validated in a past study on the detection of model synthetic surfactants (e.g., SDS and DTAB) in water^26^ and introduces several new additions to improve workflow and experimental consistency, enable higher throughput for data collection and analysis, and expand the potential reach of this approach in the context of practical applications for PFAS detection. These experimental changes include the use of an *xyz* robot to automate the fabrication of LC droplet microarrays and the collection of cross-polarized microscopy images of the microarrays at substantially lower magnifications (e.g., using a 20× objective lens). This change enabled the capture of images containing many more droplets (typically 24–30 droplets per image) simultaneously compared to higher-magnification images (e.g., obtained using a 60× objective lens, which results in only one droplet per image) used in our past study; studies described below demonstrate that the information contained in these lower-magnification images is sufficient to enable sensitive and selective detection of PFAS using ML-assisted techniques. In addition, in a significant shift from the purely supervised CNN approach we used in our past study, this current work uses a semi-supervised learning strategy. More specifically, we integrated an autoencoder network jointly trained with a classifier network while maintaining a CNN architecture similar to our earlier models for both the encoder and decoder components. Autoencoders are widely used for unsupervised learning tasks such as dimensionality reduction, feature extraction, denoising, and data generation by encoding input data into a compressed representation and reconstructing it back to its original form.^44^ This approach not only lowers computational costs but also enhances the generalizability of the model, as the latent features can be exploited for transfer learning.^44,45^ Transfer learning enables our model to perform classification tasks on previously unseen data sets, such as LC droplets treated with different amphiphiles (vide infra), allowing the application of insights gained from one set of conditions to predict others and, thereby, broadening the utility of the detection method.^46–48^ We focus on classification in this work due specifically to the goal of distinguishing between PFAS concentrations in different water matrices or between different PFAS species, which inherently involves multiple categories (i.e., the choice of water matrix).

We initiated our studies by using our new workflow to detect the presence of PFOA (chemical structure shown in Figure 1A) in various types of water samples. The workflow reported here is depicted in Figure 1B. Microcontact-printed LC arrays were treated with four concentrations of PFOA dissolved in three water matrices, including ultrapure Milli-Q laboratory water, municipal tap water, and simulated river water samples containing dissolved organic matter, resulting in an overall group of 12 classes. As described in greater detail in the Materials and Methods section, simulated river water was prepared by dissolving DOM (isolated from the Upper Mississippi River using reverse osmosis combined with electrodialysis) into samples of municipal tap water to give a concentration of 10 mg C/L, and the pH was adjusted to 7.0. The concentrations of PFOA in these samples were confirmed using an established LC–MS/MS method. The actual experimentally determined concentrations for these samples are provided in Table S2; for simplicity, we refer to these concentrations from here on as 1 ppm, 1 ppb, and 1 ppt. Cross-polarized microscopy images of the arrays were collected at 20× magnification with more than 15 trials for each condition tested, and images of individual droplets were cropped and augmented following previously established methods (adapted for multidroplet arrays and excluding images of droplets judged to be faint or substantially out of focus).^26^ All of the tested concentrations in the different water systems lead to LC droplets with apparent “bipolar” configurations^27,28,31,49,50^ that exhibited complex, multicolored optical appearances that were impossible to accurately or meaningfully differentiate from each other using the trained human eye (representative images of these droplets are shown in Figure 1C and are discussed in further detail below). The resulting tens of thousands of images of droplets were then fed into the autoencoder, which compressed these inputs into lower-dimensional representations, referred to as latent space (Figure 1B), and the decoder attempted to reconstruct the original input data. Through the process of learning to condense and restore original data inputs, the autoencoder was able to capture critical patterns and structures through the latent space, which was jointly trained with a classifier to predict the concentrations and water systems tested.

**Figure 1:**
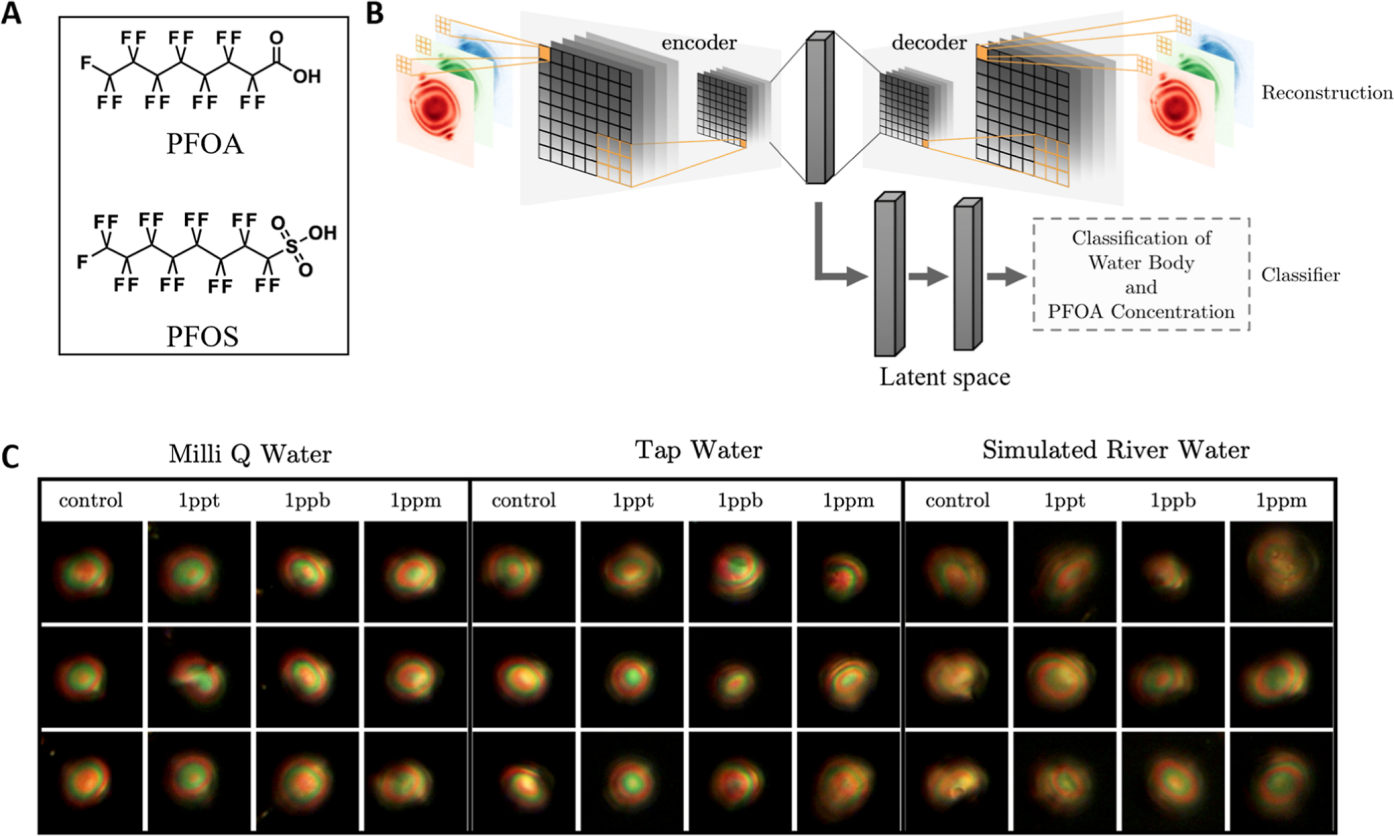
(A) Chemical structures of perfluorooctanoic acid (PFOA) and perfluorooctanesulfonic acid (PFOS). (B) Schematic showing the machine learning workflow used to analyze results arising from microcontact printed arrays treated with PFOA. The workflow involved feeding cross-polarized images of the arrays into an autoencoder, which compresses the images into lower-dimensional latent space representations and then reconstructs them. The classifier network is then trained with the latent space information for detecting PFAS. (C) Representative cross-polarized images of LC droplets at 20-fold magnification. Droplets were treated with PFOA dissolved in Milli-Q water, municipal tap water, and simulated river water at concentrations of 1 ppm, 1 ppb, and 1 ppt. All tested conditions resulted in droplets with apparent bipolar configurations that exhibited complex, multicolored patterns that were indistinguishable to the trained human eye. See Materials and Methods for additional details related to the standardization of conditions used to perform these experiments and acquire the images of LC droplets.

Representative examples of the original and reconstructed images are shown in Figure S1A. The two sets of images are generally and overall indistinguishable to the trained human eye. Inspection of these images reveals the darker defect points at opposite poles of the droplets and the colorful concentric ring patterns characteristic of LC droplets in the bipolar configuration and apparent in the original images to be successfully recreated, while some background noise was removed. The successful reconstruction of the original image inputs shows that the latent space can capture significant patterns relevant to the physical characteristics of the LC droplets, making the training of the classifier network (a simple fully connected neural network) using these condensed features promising.

The results of the 5-fold cross-validation (CV) for detecting PFOA (1 ppm, 1 ppb, and 1 ppt) in the different water systems are shown in Figure 2A, with the main diagonal indicating the percentages of correct predictions. The boxes with dotted lines in Figure 2A highlight results where the water systems in which PFOA was dissolved were correctly identified even if the specific concentrations of PFOA were not accurately predicted. Inspection of these results reveals that the algorithm was able to differentiate LC droplets treated with low concentrations of PFOA (i) from each other and (ii) from the controls with an overall prediction accuracy of around 77% (a random prediction for a 12-class classification would be around 8.3%). It is worth noting that the control samples do not contain quantifiable background PFOA based on LC–MS/MS measurements using the established protocol.^9^ This does not rule out the potential presence of ultralow levels of PFOA (e.g., at parts per quadrillion (ppq) levels)^51^ but reflects the detection limit of this current gold-standard testing method. From all of the concentrations tested, the prediction accuracy generally increased with increasing PFOA concentration (see Figure 2A). The accuracy of the predictions for the simulated river water samples (73% accuracy for 1 ppt PFOA) was slightly lower than the other two water systems (80% accuracy for 1 ppt PFOA for both Milli-Q and tap water). This result is likely due to the additional complexity of the simulated river water samples, which contain both ions present in the municipal tap water and other organic compounds present in the intentionally added DOM. Further inspection of these results reveals that samples within the same water system tend to be mispredicted with each other rather than with samples from other water systems, as indicated by the darker shades of gray coloration within the three boxes with dotted lines in Figure 2A.

**Figure 2:**
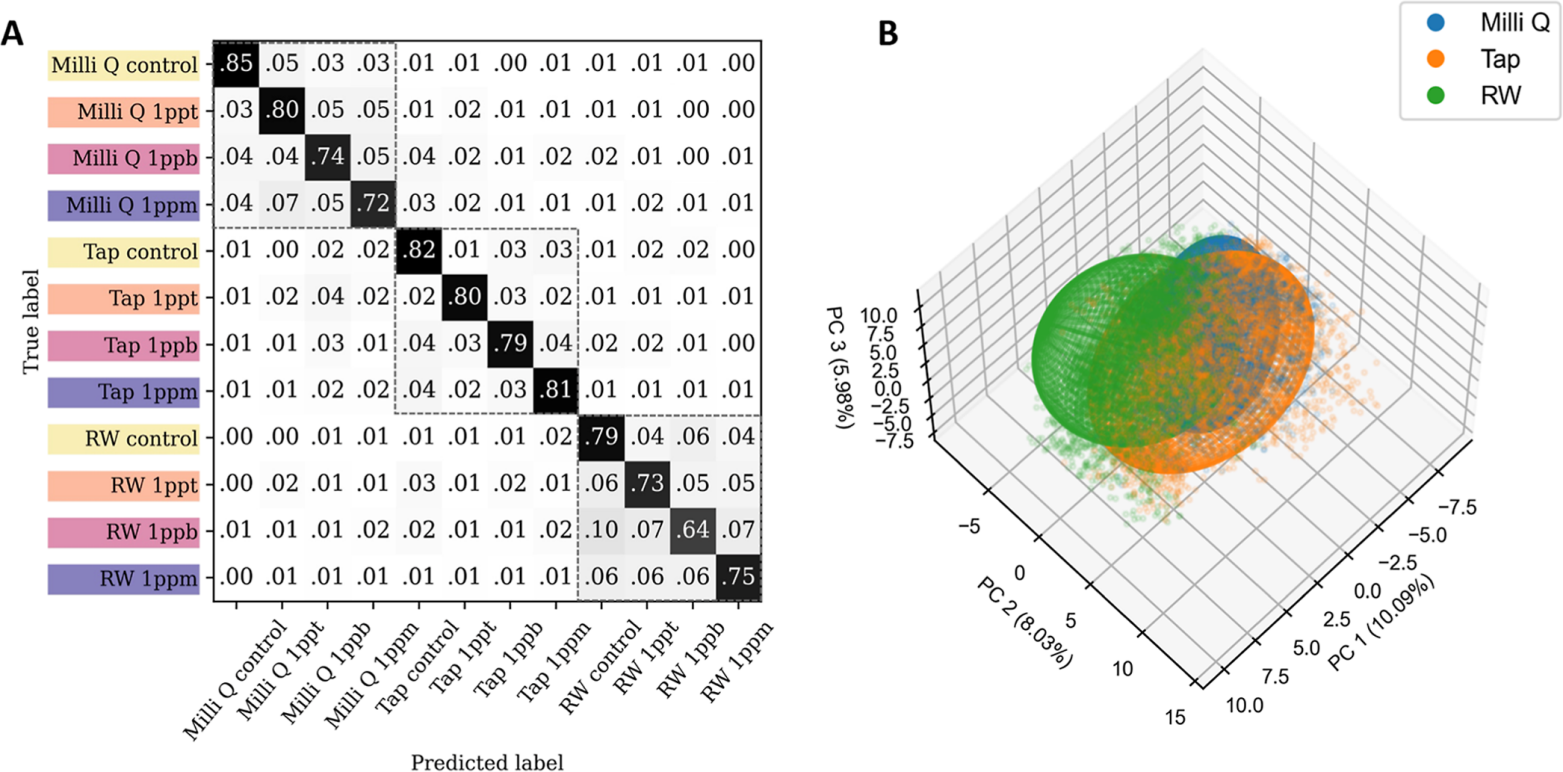
(A) Confusion matrix showing the results of the 5-fold cross-validation for detecting PFOA concentrations of 1 ppm, 1 ppb, and 1 ppt in different water systems. The PFOA concentrations are color-coded, and the water systems are included in the labels. Numerical values are colored in grayscale with different shades of gray corresponding to the numerically indicated prediction accuracy with a scale varying from white (for values equal to zero) to black (for values equal to 1). The main diagonal indicates correct predictions. The average F1 score across folds is 0.77 ± 0.01. (B) Principal component analysis (PCA) of the autoencoder’s latent features plotted in 3D space. The PCA plot of the latent features reveals distinct separation, particularly between droplets from simulated river water (RW) and municipal tap water. PCA of the input images is shown in Figure S1B for comparison. The percent variability explained for each PC is included in the axis labels.

Analysis of the latent space also revealed the effectiveness of the autoencoder. We compared the data distribution using principal component analysis (PCA) to both the input LC droplet images (Figure S2B) and the latent features trained by the autoencoder (see Figure 2B). PCA is a technique for reducing the dimensions of a data set by projecting high-dimensional data onto a lower-dimensional space. This method helps visualize and understand the distribution of data points. Generally, in the PCA plot for the input images (Figure S2B), the droplets from different water bodies are indistinguishable, supporting the observation that all droplets appear similar by visual inspection, thus making classification by conventional methods challenging. In terms of the percent variability explained, the first three PCs of the original images capture 31%, 11%, and 6% of the variance, respectively. Conversely, the PCA plot for the autoencoder’s latent features (Figure 2B) shows a certain level of distinction between droplets treated with different water samples, particularly between the simulated river samples and the municipal tap water samples. Although the latent space only captures 10%, 8%, and 6% of the variability for the first three PCs, it achieves more effective visual separation of different class categories, indicating that the learned features in the latent space are more complex and subtle, aiding in better classification despite the lower explained variance. This highlights the advantage of using an autoencoder, as it can learn more discriminative features, facilitating better differentiation between droplets that are otherwise similar in appearance. Overall, this set of experiments showcases the successful use of our approach to detect the presence of trace amounts of PFOA (ranging from 1 ppm down to 1 ppt, which is lower than the U.S. EPA regulation level for drinking water) in various complex water systems. By jointly training a classifier on the latent space of an autoencoder rather than purely on the raw input data (as in a plain CNN classification approach), the model is more robust to variations and noise and mitigating overfitting since the latent space representation captures only the essential underlying structures.

We next focused on understanding the extent to which this approach could be used to selectively discriminate between LC droplets treated with PFOA and LC droplets treated with PFOS. As shown in Figure 1A, the structures of PFOA and PFOS differ only in their head groups; this small structural difference renders it difficult to differentiate or simultaneously detect these compounds using many testing methods developed for on-site testing.^19^ We previously reported that the combination of these LC arrays with machine learning analysis can enhance sensing selectivity for droplets treated with either SDS or DTAB, two synthetic surfactants that have the same tail length but different head groups.^26^ We hypothesized that the algorithm used here would also be able to pick up on nuanced and otherwise visually unapparent differences in the optical outputs of LC droplets resulting from the adsorption of PFOA or PFOS. We dissolved PFOS in simulated river water to a concentration of 3.5 ppt (stock concentrations confirmed by LC–MS/MS, see the Materials and Methods section for details). To further challenge the system and mimic more complex and realistic scenarios in which mixtures of multiple PFAS exist, we also created a sample comprising a mixture of PFOA (1 ppt) and PFOS (3.5 ppt) in simulated river water. Representative images of LC droplets treated with these water samples are shown in Figure 3A. Visual inspection of these images reveals that all droplets exhibit indistinguishable optical features. For this part of the study, in view of the similarities in the optical outputs of LC droplets treated with simulated river water samples containing PFOA and PFOS, we applied transfer learning to determine whether it was possible to leverage the existing pretrained model based on PFOA data, as described above, to extract features of PFOS-related data and selectively predict the presence of each and their mixture.

**Figure 3:**
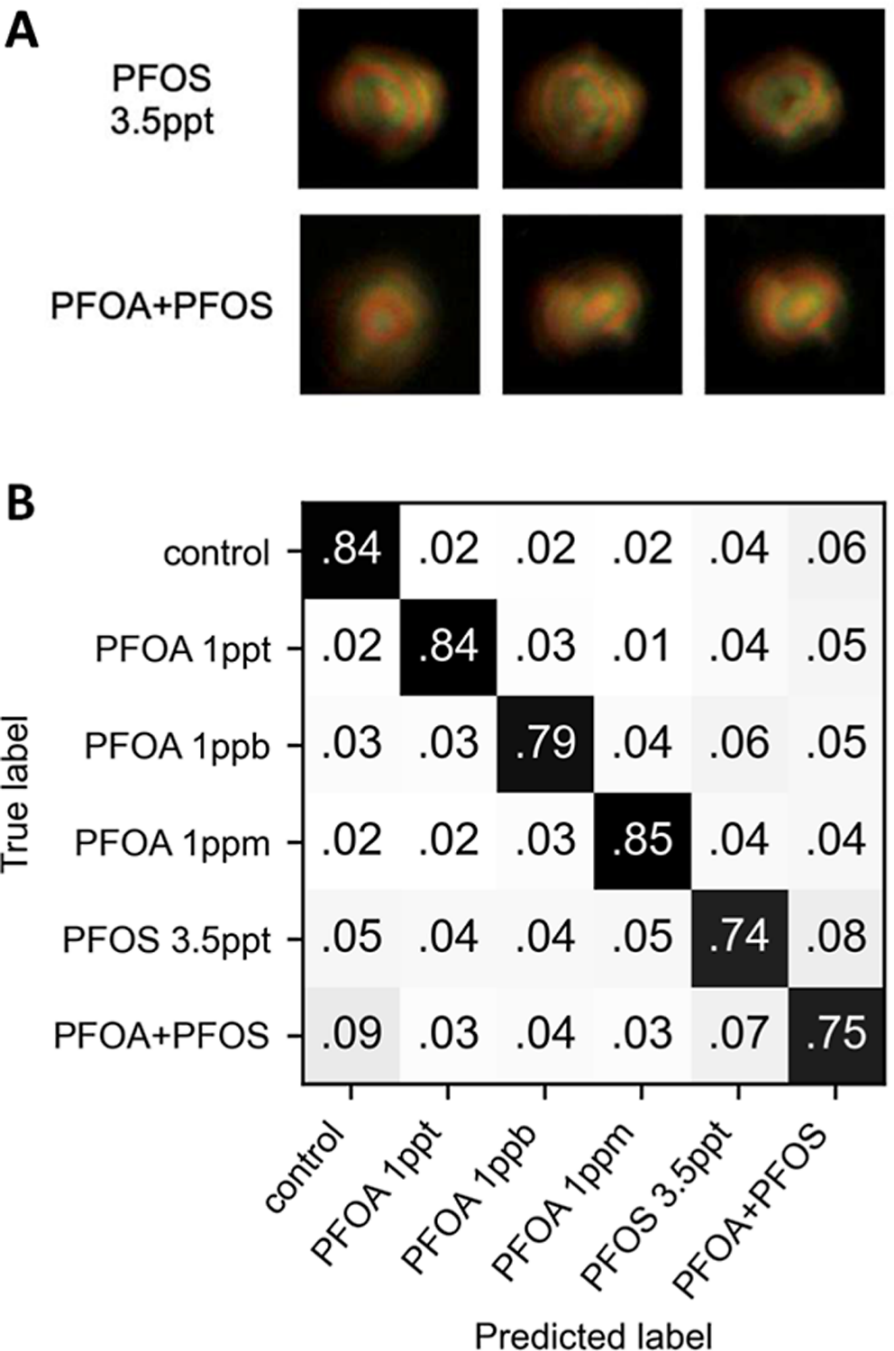
(A) Representative images of LC droplets treated with PFOS (3.5 ppt) and a mixture of PFOA (1 ppt) and PFOS (3.5 ppt) in simulated river water. The droplets exhibit bipolar configurations and are visually indistinguishable from each other. (B) Confusion matrix showing results of the 5-fold cross-validation of the classifier, which analyzed latent space information extracted from the pretrained autoencoder using transfer learning techniques to differentiate between PFOA (at various concentrations), PFOS, and their mixture in simulated river water. The average F1 score across folds is 0.80 ± 0.01.

Optical images of droplets from arrays treated with PFOS (3.5 ppt) and the PFOA (1 ppt) + PFOS (3.5 ppt) mixture were fed through the pretrained model (encoder), and latent space representations were then generated to train a new classifier to differentiate between PFOA (at various concentrations), PFOS, and their commixture in simulated river water. The 5-fold cross-validation result is shown in Figure 3B, with the diagonal line showing the percentages of correct predictions. The overall prediction accuracy was around 80%. This accuracy is significantly higher than a random guess, which would have an accuracy of around 16.7% for this 6-class classification. Based on the prediction accuracy of each condition, it is evident that this system can differentiate river water controls from river water containing either PFOA, PFOS, or a PFOA + PFOS mixture. It also retains the ability to report quantitative information about the concentration of PFOA. Another observation from the results shown in Figure 3B is that samples containing PFOS are more likely to be predicted as a mixture of PFOA and PFOS than as different concentrations of PFOA, indicating some shared features between the two 3.5 ppt PFOS and PFOA + PFOS mixture conditions. However, these results also highlight a current limitation of this approach. Given that the classes in Figure 3B correspond to solutions with a range of ppm, ppb, and ppt PFAS concentrations that differ by orders of magnitude, samples misclassified here correspond to substantial errors in PFAS concentration. Fundamentally, this classification approach relies on having a sufficient amount of data across a sufficient number of categories to accurately classify differences in categories of concentration; in general, with more training data, classification errors should become smaller as the number of categories increases to approximate a more continuous range of concentration. In this context, we note that our past study on the classification of concentrations of synthetic surfactants using this classification approach^26^ suggests that increasing the amount of training data to include a wider range of PFAS concentrations should also lead to improved accuracy for the prediction of PFAS concentration, providing a path toward further model improvement. Overall, these results demonstrate that transfer learning can be used to introduce new and useful levels of selectivity for the detection of PFAS using this approach and highlight the need for substantially larger sets of training data to further develop the model prior to deployment in real-world settings.

Compared with regression models, the classification model we used here predicts the discrete categories we tested and, as a result, has limited capacity to report the exact or actual concentrations of the PFAS in a given unknown sample. We note that a regression-based approach to the prediction of PFAS concentrations in a single water source could also be developed using our LC array-based approach by gathering training data using a much larger and more continuous range of individual PFAS concentrations rather than the discrete concentrations used in this study. However, considering that the U.S. EPA has recently set the non-enforceable maximum contaminant level goals (MCLGs) for both PFOA and PFOS to be zero,^15^ a sensing system that can simply discern samples contaminated with PFAS from samples of clean water free of PFAS should also be highly beneficial in practical scenarios. Thus, in the next part of our study, we focused on training the model to recognize characteristic features of LC droplets exposed to various PFOA concentrations and to distinguish these samples from control samples across all three water systems. For these studies, we used a binary classification approach to train the classifier. For samples prepared using Milli-Q and tap water, data for all concentrations of PFOA tested (1 ppm, 1 ppb, and 1 ppt) were combined into one group (labeled as “PFAS”), and data obtained using water controls was used as the other group (labeled as “Control”). For samples prepared using simulated river water, data for all concentrations of PFOA (1 ppm, 1 ppb, and 1 ppt), 3.5 ppt PFOS, and 3.5 ppt PFOS +1 ppt PFOA mixtures were grouped into one class (labeled as “PFAS”), and data obtained using water controls was used as the other group. The resulting class-wise accuracies are summarized in the bar plot shown in Figure 4A.

**Figure 4:**
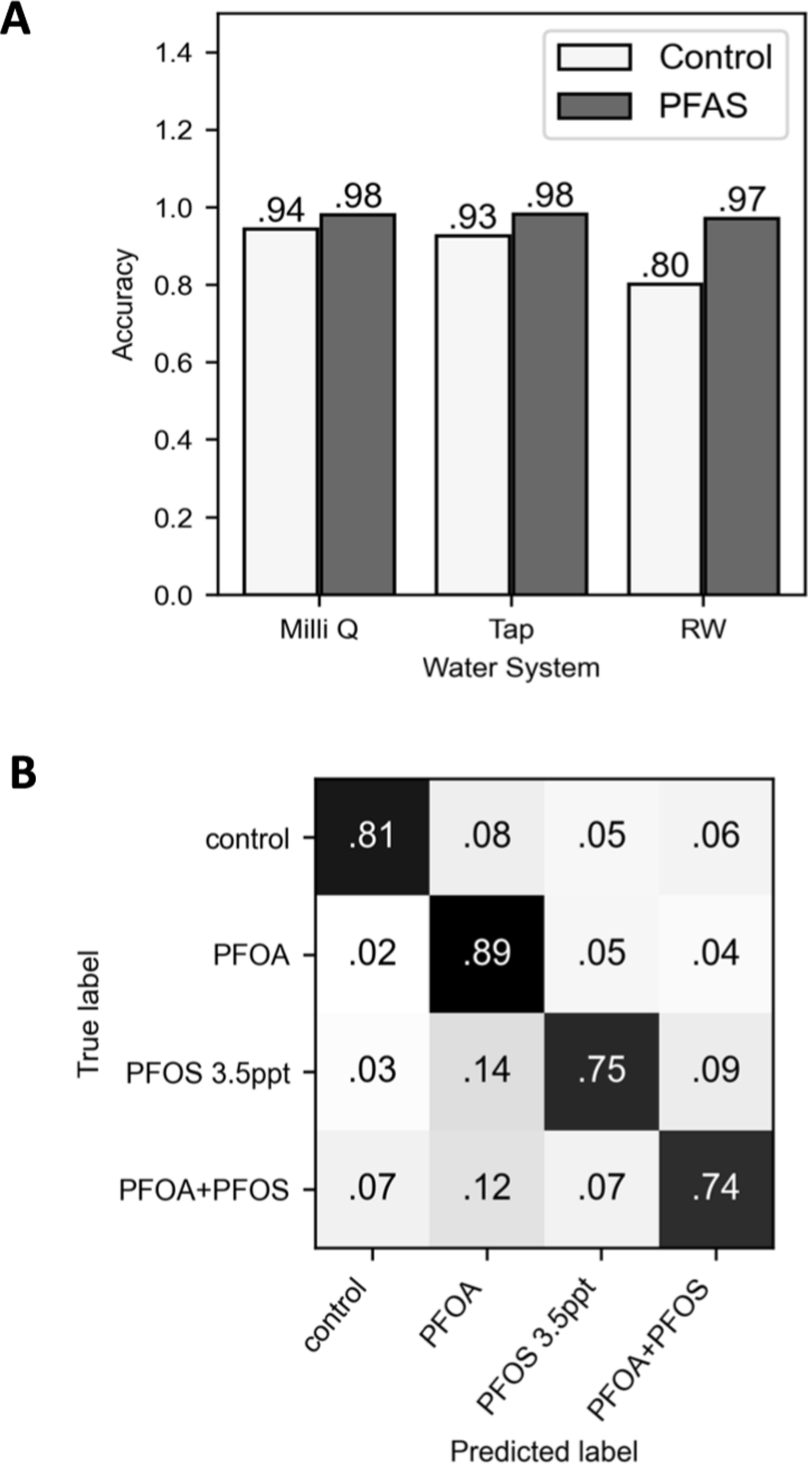
(A) Bar plot summarizing the class-wise accuracies of the binary classification model for detecting the presence of PFAS. The model was trained to differentiate between controls and PFAS-treated samples across three different water systems. (B) Confusion matrix showing multiclassification results for simulated river water samples distinguishing between PFOA, PFOS, a PFOA + PFOS mixture, and controls. The average F1 score across folds for 5-fold cross-validation is 0.80 ± 0.01.

For the Milli-Q and tap water systems, the algorithm successfully predicted the presence of PFOA with 97.2% and 96.8% accuracy, respectively. For simulated river water, the prediction accuracy for the presence of PFAS was 94.3%. For all three water systems tested, the overall prediction accuracies all exceeded 90%, demonstrating that the classifier was able to find common features in the latent space representations of varying concentrations of PFAS and use them to differentiate from the controls. We also analyzed the data distribution using PCA on the latent features extracted (Figure S2) for LC droplets treated with controls and PFAS solutions in the three different water systems tested. The 3D PCA plot for the latent features shows a certain level of distinction between LC droplets treated with controls and those treated with PFAS, particularly for simulated river water samples, as illustrated in Figure S2. This again suggests that, even though the raw images of LC droplets treated with controls and those treated with PFAS are, in general, indistinguishable to the trained human eye, the introduction of PFAS did lead to subtle changes that could be identified by the autoencoder. We note that, because the simulated river water was prepared by adding dissolved organic matter to tap water, it has many additional species that PFAS molecules could potentially bind or adsorb to through electrostatic or hydrophobic interactions^38,52^ to form complexes or consume free PFAS molecules that could change the nature of interactions with LC droplets. It is thus reasonable to assume that PFAS in simulated river water could trigger changes in the optical outputs of LC droplets that are different from those in other environments that do not contain these additional species or, potentially, other species or assemblies that could result from those interactions.^28,53^ We also note that the workflow reported here focused on learning the differences between a control sample and a sample spiked with a certain known amount of PFAS. If the unspiked control water samples already contain some amount of PFAS, the algorithm used here would not be able to predict it. Overall, the specific approach described here may be better suited for monitoring changes or differences in PFAS levels in comparison to other control samples than it is for predicting specific concentrations of PFAS in a single given water sample.

Because knowing the specific species or structure of PFAS present in a water sample is also important for health risk evaluation and the identification of proper environmental remediation strategies, we also characterized the ability of this system to not only sense the presence of PFAS but also predict the identities of different species present in the water. For this set of experiments, we divided the simulated river water samples used in the experiments described above into four categories. All concentrations of PFOA were grouped together and compared with controls, PFOS, and PFOA + PFOS mixture samples. We note that we utilized a stratified cross-validation approach and weighted loss function, as detailed in the Materials and Methods section, to avoid inaccuracies associated with the larger number of PFOA samples in this data set. The result of this multiclassification is shown in Figure 4B. The average prediction accuracy was around 80.2%, which is significantly higher than that of a random guess, which would have a 25% accuracy for this 4-class classification, highlighting the success of this approach in discerning PFOA, PFOS, and the mixture of the two. Close inspection of the 5-fold cross-validation results reveals that PFOA samples with differing concentrations are more likely to be predicted as samples with PFOS or the PFOA + PFOS mixture than as controls. Also, samples with PFAS tend to be mispredicted among each other in comparison to controls. This suggests some common features observed by the algorithm among LC droplets treated with PFOA and PFOS. It is also worth noting that most mispredicted droplets were categorized as PFOA samples; this result could arise, at least in part, from the fact that the size of the PFOA data set used in this analysis was approximately two times larger than the data sets used for the other three categories.

To further confirm that our ML approach is observing and using physicochemically relevant features to make predictions (as opposed, for example, to simply memorizing random features in the images), we randomly assigned images of droplets treated with simulated river water only (a total of 56,600 droplet images after augmentation) into an arbitrarily labeled PFAS-containing group (28,300 droplets; 50% of the total droplets) and a control group labeled correctly as water (28,300 droplets; the remaining 50% of the droplets) and then evaluated prediction accuracies. The resulting 5-fold binary classification accuracies were around 50%, matching that of a random guess for a binary classification. This is consistent with our expectation that since all droplets in this experiment were treated with simulated river water only and the labels were randomly and arbitrarily assigned as either water or PFAS (and, thus, there were no actual differences between the two groups), the algorithm should not be able to correctly predict their specific labels. We also conducted experiments to collect an additional test data set containing images that were not used in model training and/or validation to conduct a blind test; these experiments were conducted at varying concentrations of PFOA (1 ppm, 1 ppb, 1 ppt), PFOS (3.5 ppt), and PFOA (1 ppt) + PFOS (3.5 ppt) mixtures in simulated river water. We then analyzed this test set using both the binary classification model (to differentiate controls from PFAS) and the multiclassification model (to predict the specific species of PFAS present). The resulting prediction accuracies were comparable to the 5-fold classification results reported in Figure 4A,B. The binary classification model predicted the presence of PFAS with more than 95% accuracy, and the multiclassification model had an overall prediction accuracy of 87%. This result further validates the predictive power of our system and demonstrates the potential application of our combined experimental/ML workflow to monitor and report on PFAS contamination in complex water matrices.

## Summary and Conclusions

In conclusion, we have reported new approaches to the machine learning-assisted analysis of water-treated LC droplet microarrays for the sensitive and selective detection of environmental PFAS pollutants. We developed an autoencoder neural network to capture critical features of LC droplet arrays treated with water samples containing different concentrations of PFOA and then used the output latent space to train a simple classifier network. Our results demonstrate that this approach can be used to report accurately on PFOA across a broad range of concentrations (1 ppm, 1 ppb, and 1 ppt) even though differences in the optical outputs of the droplets themselves are visually indistinguishable to the trained human eye. Our results also demonstrate that it is possible to detect these low concentrations of PFOA in multiple water systems of varying composition and complexity, including simulated river water containing inorganic ions and dissolved organic compounds that have the potential to interfere with or change the nature of the interactions between the LC droplet microarrays and PFAS. We also used a transfer learning approach to introduce selectivity to the system, enabling the identification and differentiation of samples containing PFOA, PFOS, or PFOA + PFOS mixtures. Finally, our results provide a proof-of-concept demonstration of a simplified sensing process focused on detecting either the presence or the absence of PFAS relative to controls and the specific species of PFAS present for potential practical applications. The use of an autoencoder to extract latent space simplifies the transfer learning process, making the method more generalizable and enabling the system to be trained to be compatible with other water systems or potentially for use in detecting other types of environmental pollutants. Although the proof-of-concept results presented in this work currently lack the quantitative accuracy necessary for real-world deployment, particularly in more complex water sources that could also contain numerous other potentially interfering species, we anticipate that model accuracy can be further improved through a substantial increase in experimentally obtained training data. Similarly, while the approach we report here considers fundamental questions more well suited to classification, such as the potential to discriminate between PFAS samples in complex, real-world water matrices and to discriminate between different PFAS structures and mixtures of PFAS, the collection of additional experimental data for a broader range of PFAS concentrations could further enable the extension of this approach to the prediction of more continuous PFAS concentrations.

## Supplementary Material



## Data Availability

All data and scripts needed to reproduce the results can be found at http://doi.org/10.5281/zenodo.15609463.

## Notes

Additional information including plots, images, and analytical data related to the quantification and benchmarking of PFAS concentrations in water using standard LC–MS/MS methods (PDF)
