# Supplementary material for: A Machine Learning-Assisted Liquid Crystal Droplet Array Platform for the Sensitive and Selective Detection of Per- and Polyfluoroalkyl Substances (PFAS) in Water
Source: ACS Sens. 2025 Sep 25;10(10):7343–53. doi: 10.1021/acssensors.5c00907 (PMC12560129; doi:10.1021/acssensors.5c00907)
Supplement: Supplementary file 1 [file se5c00907_si_001.pdf]

A Machine Learning-Assisted Liquid Crystal Droplet Array Platform for the Sensitive and Selective Detection of Per- and Polyfluoroalkyl Substances (PFAS) in Water

Fengrui Wang,[1,¶] Shiyi Qin,[2,¶] Zhao Yang,[3] Leena M. Edwards-Medina,[4] Benjamin L. Chiu,[2] Claribel Acevedo-Vélez,[4] Christina K. Remucal,[3,*] Reid C. Van Lehn,[2,*] Victor M. Zavala,[2,5,*] and David M. Lynn[1,2,*]

[1]Dept. of Chemistry, Univ. of Wisconsin–Madison, 1101 University Ave., Madison, WI 53706, USA; [2]Dept. of Chemical and Biological Engineering, Univ. of Wisconsin–Madison, 1415 Engineering Dr., Madison, WI 53706, USA; [3]Dept. of Civil and Environmental Engineering, Univ. of Wisconsin–Madison, 660 North Park St., Madison, WI 53706, USA; [4]Dept. of Chemical Engineering, University of Puerto Rico-Mayagüez, Call Box 9000, Mayagüez, PR 00681-9000, USA; [5]Mathematics and Computer Science Division, Argonne National Laboratory, 9700 S. Cass Ave, Lemont, IL, 60439, USA; [¶]These authors contributed equally to this work. Email: (C.K.R) remucal@wisc.edu, (R.C.V) vanlehn@wisc.edu, (V.M.Z.) victor.zavala@wisc.edu, (D.M.L.) david.lynn@engr.wisc.edu

Supporting Information

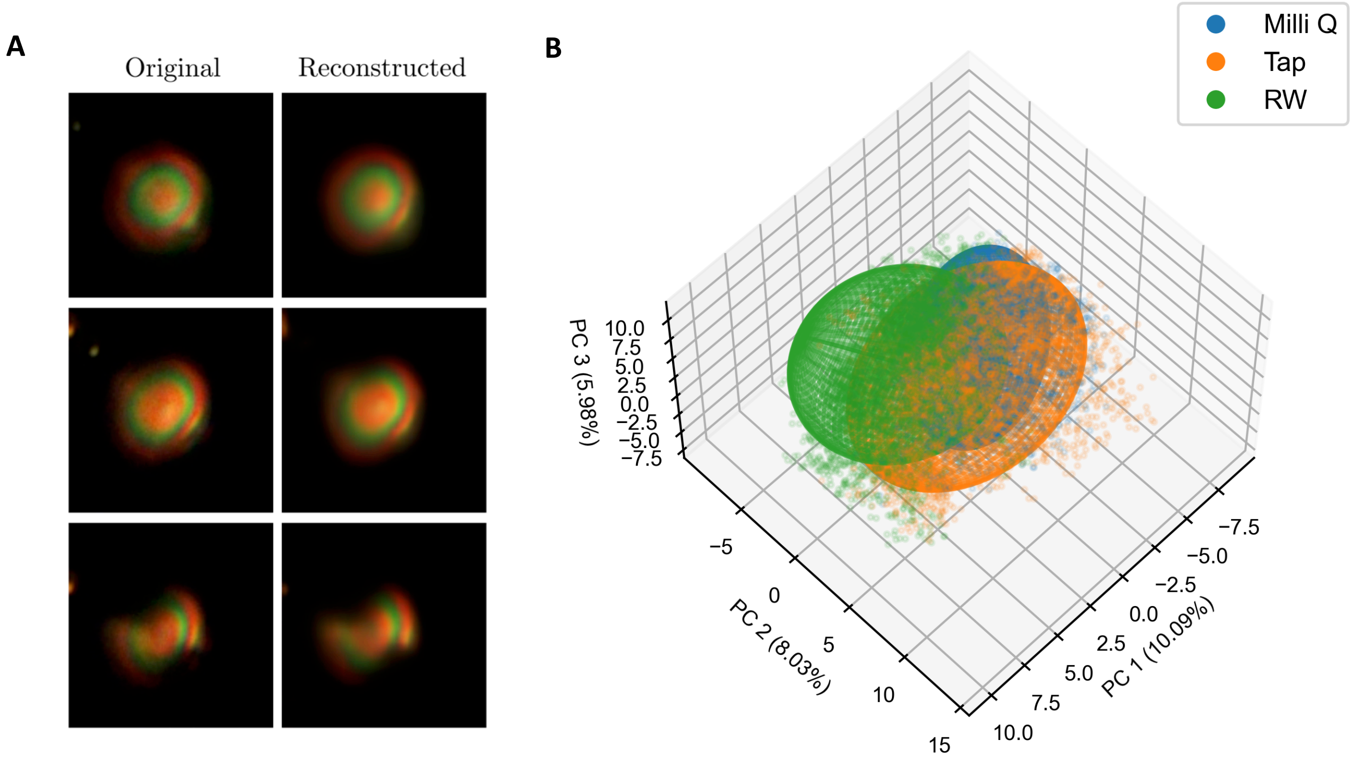

Figure S1. A) Representative images of original and reconstructed liquid crystal (LC) droplets for samples from Milli-Q water, municipal tap water, and simulated river water (RW). The reconstructed images are, overall, visually indistinguishable from the originals, successfully recreating defect points at opposite poles of the droplets and the colorful concentric ring patterns while reducing some background noise. B) Principal component analysis (PCA) of the original LC droplet images. The PCA plot demonstrates that droplets from different water systems are generally indistinguishable, supporting the visual observation that all droplets appear similar and highlighting the challenge of classification based purely on raw image data.

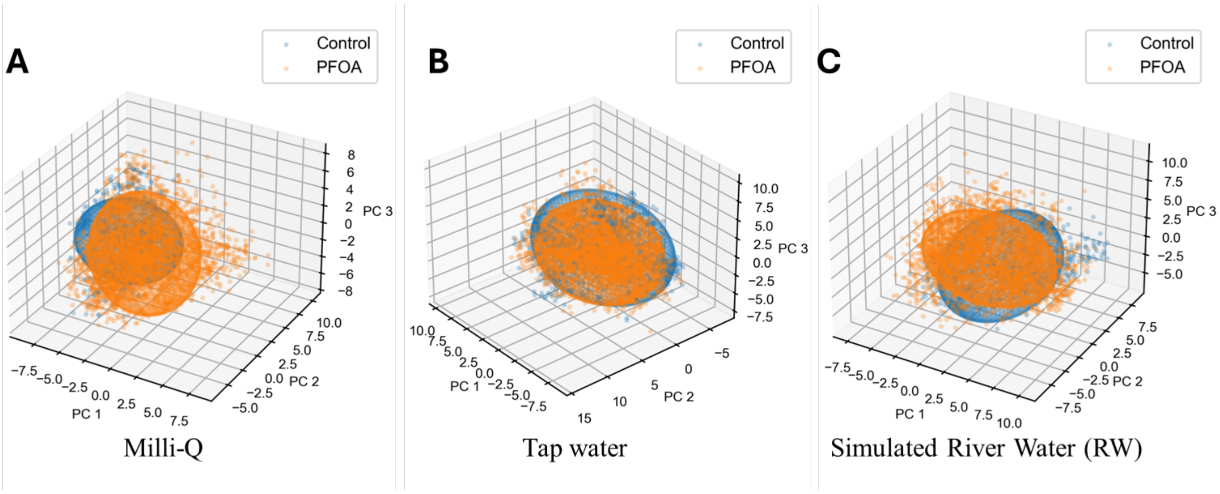

Figure S2. 3D Principal Component Analysis (PCA) plots of latent features extracted from LC droplet images treated with controls and PFAS solutions across three different water systems (MilliQ water (A), municipal tap water (B), and simulated river water (C)). The PCA plots show a certain level of distinction between LC droplets treated with controls and those treated with PFAS, particularly for simulated river water samples, indicating that the introduction of PFAS leads to changes in hidden structures captured by the autoencoder.

Table S1. Information on multiple reaction monitoring (MRM) about transitions (quantifier and qualifier) and retention times (RT) of PFOA and PFOS monitored during this study. For more details of the LC-MS/MS procedures, see the Materials and Methods section and previous publication.[1]

**Analyte**	**Precursor** *m/z*	**Quantifier** *m/z*	**Qualifier** *m/z*	**RT (min)**
PFOA	413	368.9766	168.9894	8.89
$^{13}C_8$ PFOA	421	376.0001		9.89
PFOS	499	79.9574	98.9558	10.57
$^{13}C_8$ PFOS	507	79.9574		10.57

Table S2. PFAS quantification for selected samples used in this study using LC-MS/MS.

Sample Label	Concentration Determined
0.1 mg/mL PFOA	0.10 ± 0.02 mg/mL
1 ppm PFOA	1.04 ± 0.14 ppm
1 ppb PFOA	0.83 ± 0.14 ppb
Milli-Q	0 ppt PFOA; 0 ppt PFOS
Municipal Tap Water	0 ppt PFOA; 0 ppt PFOS
Stock PFOS	1.39 ppb

Additional information related to the preparation and analysis of these samples can be found in the Materials and Methods section. The "Concentration Determined" column shows the LC-MS/MS-determined concentrations, with standard deviations, of PFOA and PFOS in the water samples used in this study. As described in the main text, samples containing PFAS were prepared at target concentrations of 1 ppm, 1 ppb, and 1 ppt, as indicated in the "Sample Label" column. The measured concentrations differ from these target concentrations, and those actual measured values are shown in the "concentration determined" column. For simplicity in the main text, we refer to these solutions by their target concentrations (or "sample labels") rather than their actual measured concentrations; those measured concentrations are provided here for reference. For the Milli-Q and municipal tap water samples, the measured concentrations are labeled as "zero" because the concentrations of PFOA and PFOS were below the limit of quantification (LOQ) of the instrument.

Table S3. Volumes used for solid phase extraction (SPE) and corresponding limits of detection (LOD) and quantification (LOQ) for extracted samples.

Sample	Volume for SPE (mL)	LODs for PFOA and PFOS (ppt)	LOQs for PFOA and PFOS (ppt)
Municipal Tap Water	992	0.03	0.10
Simulated River Water	217	0.15	0.46
Milli-Q	505	0.07	0.20
Spiked sample (ppt level)	500	0.07	0.20

Supporting Information References

1. Balgooyen, S.; Remucal, C. K. Impacts of Environmental and Engineered Processes on the PFAS Fingerprint of Fluorotelomer-Based AFFF. *Environmental Science & Technology* **2023**, *57* (1), 244-254.
